# Corporate Profits and the Health of Americans

**DOI:** 10.3390/healthcare14010119

**Published:** 2026-01-04

**Authors:** Anthony Biglan, Ronald J. Prinz, Diana H. Fishbein

**Affiliations:** 1 Oregon Research Institute, Eugene, OR 97403, USA; 2 The National Prevention Science Coalition to Improve Lives, Chapel Hill, NC 27599, USA; 3 Research Center for Child Well-Being, University of South Carolina, Columbia, SC 29208, USA; 4 Frank Porter Graham Child Development Institute, University of North Carolina, 910 Raleigh Road, Chapel Hill, NC 27599, USA; 5 Human Development and Family Studies, The Pennsylvania State University, State College, PA 16802, USA

**Keywords:** harmful marketing, healthcare system, health disparities, Medicare, premature mortality, prevention, profit maximization, wellbeing

## Abstract

A large and growing empirical literature documents that privatization, deregulation, financialization, and under-regulation of harmful industries are associated with adverse health outcomes in the United States. However, this evidence remains fragmented across sectors and rarely articulates a unifying causal framework. This paper advances the literature by integrating findings across health care, harmful-product industries, and economic and social policy to demonstrate that **corporate profit maximization functions as a cross-cutting driver of health disparities and premature mortality in the United States.** We synthesize evidence showing that profit-driven incentives shape insurance markets, hospital and physician practice ownership, pharmaceutical marketing, and the aggressive promotion of tobacco, alcohol, ultra-processed foods, opioids, firearms, and fossil fuels—together contributing to more than one million deaths annually. We further document how corporate influence over public policy has increased poverty, economic inequality, and discrimination, all of which are powerful social determinants of health. In contrast to sector-specific analyses, this paper presents a unified, systems-level account of how profit-first governance undermines population health. We conclude by describing how a social movement to achieve a single payer system that provides Medicare for All would not only vastly improve public health, it would be a catalyst for numerous other reforms that enhance the general wellbeing.

## 1. Introduction

In earlier work by Biglan, Prinz, and Fishbein [1], we argued that preventing illness and premature death requires addressing social and economic policies that produce poverty, discrimination, and other social determinants of health. Recent studies strengthen this argument by showing how the financialization and marketization of U.S. health care contribute directly to widening health disparities and avoidable mortality. Large quasi-experimental studies demonstrate, for example, that hospital privatization increases short-term mortality among Medicare patients [2], private equity acquisition of nursing homes raises rates of hospitalization and death [3], and insurance-company delays or denials of care can lead to preventable deaths [4,5]. More broadly, private equity firms are financial actors whose business model is to maximize investor returns by buying, reshaping, and reselling companies—often on relatively short timelines. Needless to say, this model presents challenges to quality health care and public well-being.

Together, these findings reflect a broader pattern: profit maximization has become a dominant organizing principle in U.S. health care. Its influence extends beyond health care delivery itself, as corporations also market harmful products—including tobacco, alcohol, ultra-processed foods, opioids, firearms, and fossil fuels—that collectively cause more than a million deaths annually. Corporate power has likewise shaped tax, labor, regulatory, and safety-net policies in ways that increase poverty and economic insecurity, both well-established drivers of chronic disease and premature mortality.

## 2. The Sixty Year Ascendancy of Profit Maximization

In 1970, Milton Friedman published what has come to be called the Friedman Doctrine; “The Social Responsibility of Business is to Increase its Profits” [6]. In the ensuing years, significant sectors of the business community organized to advance this view [7,8]. The result has been that corporate leaders focus almost exclusively on maximizing profits. This has corrupted many organizations to engage in profitable practices irrespective of the potential for harmful consequences. And thanks to effective advocacy by the business community and lobbyists, they face minimal regulation. In the present paper we document this fact as it applies to the health care system and the health of Americans.

## 3. The Health of Americans

The U.S. is 47th in life expectancy, far behind any other economically developed country. Bor et al. [9] compared the death rate of Americans from the 1930s to 2020 with the rates in 21 other wealthy nations. In the 1980s the American death rate began to exceed that for other countries and, over the years, has increasingly diverged from that of comparison countries.

These statistics obscure the disparities in health and longevity in the U.S. population. A 2025 report from the National Vital Statistics Reports estimates the longevity of Americans as follows: Asian 84.4, Hispanic 80, White Non Hispanic 77.5 Black 72.8, and Native American 67.8 [10]. Research shows that income and socioeconomic status are linked not only to mortality but also to physical and mental health outcomes, such as obesity, diabetes, heart disease, depression, and overall health status. Lower income and greater inequality are associated with higher rates of such health conditions, and these associations persist even after accounting for other factors (e.g., gender, race/ethnicity, region) [11].

There are three ways that the singular focus on profits has contributed to poor health. First, the health care system has evolved many profitable practices that adversely impact health. Second, the success of certain business interests in minimizing regulation has enabled companies to market products that contribute to chronic disease and premature death. Third, the success of corporations in dominating policymaking has increased poverty, economic inequality, and discrimination, all of which contribute to chronic illness and premature death [12].

## 4. How Profit Maximization in Health Care Undermines Health and Contributes to Premature Death

### 4.1. The Financialization of Health Care

In describing the financialization of health care in the U.S. Bruch et al. [13] document how “a series of regulatory and policy changes empowered financial actors to make the U.S. health care system a core part of their growth strategies” [p 178]. They cite over 8000 transactions in which private equity firms purchased hospitals, nursing homes, hospice facilities, physician practices, dialysis clinics, and other health care entities, such as the buildings where health care is located. Thus, profit maximization has become the central goal of major players. Bruch et al. call for “a new wave of research” to examine the degree to which financialization is harming health. The discussion below begins to address this need.

### 4.2. The Pharmaceutical Industry

Many pharmaceutical companies have developed practices that fit this definition. There have been hundreds of thousands of opioid deaths [14] resulting from the marketing of opioids that industry leaders, such as Purdue Pharma, claimed were not addictive [15]. Pharmaceutical companies are also harming Americans by promoting the over-prescribing of medications for hyperactivity [16], antibiotics [17], and depression [18].

Failure to prevent these harms stems from the erosion of regulatory practices by agencies originally designed to prevent harm. For example, Donald Light and colleagues [19] documented corruption in the Food and Drug Administration’s (FDA) evaluation of drugs. Thanks to the industry’s lobbying and political influence, the FDA has inadequate resources to regulate industry practices and has established user fees for which the industry pays the FDA to assess the efficacy and harm of drugs. This arrangement has led to a system whereby the industry can change the molecular structure of an existing drug such that when its patent protection expires, they can substitute the new formula and prevent competition from generic drug makers. Few of the newly approved drugs provide more benefit than existing drugs, and as many as 15% risk more harm than benefit [19]. Light et al. concluded, “Despite the small number of clinically superior drugs, sales and profits have soared as marketing persuades physicians to prescribe the much more costly new products that are at best therapeutically equivalent to established drugs.”

Corruption in the pharmaceutical industry has likely contributed to its greater profitability relative to comparable industries. Between 2000 and 2018, annual profit margins of pharmaceutical companies were significantly greater than those of S&P 500 companies. Net income margin was 13.8% for the Pharma companies versus 7.7% for 357 companies in the S&P 500 [20].

### 4.3. Health Insurance

When the Obama Administration sought to establish a health care system that would provide health insurance for everyone, the Administration rightly believed it would be impossible to achieve health care reform unless the health insurance companies could profit. As a result, the Affordable Care Act (ACA) has been quite profitable for these companies. In 2022, the four largest health insurance companies had profits totaling more than $41 billion. The CEOs of these organizations reap much of the benefit of these profits. The seven most highly paid CEOs of health insurance companies made a total of $136.7 million in 2022 [21]. The bulk of their income is derived from stock awards. The pressure to maximize profits has encouraged companies to buy back their own stock to raise the price of the stock. In 2023, the seven largest health insurance companies spent $26.2 billion on buybacks—a practice that had no discernable benefit for the health of those they insured [13]. If buybacks were prohibited and the practices that delay or deny coverage were properly regulated, those funds could be used to increase coverage.

Unlike other nations, 30% of the health care costs in the U.S. pays for administrative functions incurred by providers and insurers who are forced to contend with multiple insurance plans, each with their own rules for reimbursement and advanced approval for many services [22].

### 4.4. Private Equity

The focus on profits has brought a new player onto the field. Private equity firms have begun buying nursing homes, hospice facilities, emergency rooms, and rural hospitals [23,24]. As they have done in other industries, these companies seek to maximize profits by cutting costs. An example is the closing of rural hospitals [25], which has left some communities without any hospitals.

Hospitals purchased by private equity firms have higher rates of adverse events such as falls or infections. Compared with hospitals not acquired by private equity firms, hospitals purchased by such firms experienced a 25.4% increase in Medicare patient adverse events after private equity acquisition [26].

Private equity purchases of physician practices are associated with significant increases in the cost of services, especially when private equity owns 30% or more of the practices in the community [27]. There has been little anti-trust scrutiny of these acquisitions.

One might hope that the increasing profitability of the health care system would lead to improved health of Americans. However, the opposite is true. Beyond deleterious pharmaceutical marketing, deaths can be attributed to infections that patients develop in hospitals [28], the unavailability of health insurance [29], and greater premature death linked to racial disparities [3,30].

In sum, the system we have evolved to address the health of Americans carefully monitors profits but does not even minimally track its impact on health.

### 4.5. Stress Among Health Care Providers

Our health care system also poses challenges for providers. The pandemic was especially stressful [31]. However, studies conducted before the pandemic indicated that physicians and nurses reported higher rates of stress-related problems than people in other professions. Medical students, residents, and early career physicians also reported high levels of stress [32]. An analysis of the cost of stressful conditions to the health care system estimated that there are “approximately $4.6 billion related to physician turnover and reduced clinical hours attributable to burnout each year [33]. Mergers and acquisitions are among the factors contributing to providers’ stress [34]. The uncertainty, loss of autonomy, increased workload, threat of layfoffs and other factors created by mergers and acquisitions are among many of the conditions that arise for providers.

## 5. How Profit Maximization in Other Sectors Undermines Health

### 5.1. The Marketing of Unhealthful Products

The health care system’s prioritization of profits is not the only way that corporations contribute to premature death in the U.S. In addition to the deaths that pharmaceutical marketing is causing, the marketing of cigarettes results in 480,000 premature deaths a year [35]. Alcohol use contributes to 140,000 deaths [36] and the alcohol industry markets heavily to youth. The intensive marketing of unhealthful foods to children normalizes harmful eating habits, contributing to more than 500,000 deaths per year [37]. The highly successful marketing of guns in the U.S. contributed to more than 48,000 deaths in 2021 [38], and firearms are the leading cause of death among children and adolescents since 2020 [39].

### 5.2. The Impact of Economic Policy on Health

Beyond the actions of corporations that directly undermine health, corporate control of policymaking has increased the prevalence of adverse conditions, such as poverty, economic inequality, and discrimination [1]. Numerous policies adopted over the past 50 years have increased family poverty. Table 1 lists policies that play a significant role in sustaining poverty in the U.S.

The American Community Survey data from 2022 showed that the poverty rate among those under 18 was 16.3%, which is 3.7% higher than the overall rate [49]. This rate is higher than that of 33 other members of the Organization for Economic Cooperation and Development (OECD) [50]. Although poverty among the elderly has declined significantly thanks to the indexing of social security to inflation and the creation of Medicaid, “the poverty rate among children remains high, and grew much more in 2020 compared to the poverty rate for older Americans” [51].

Child poverty is a risk factor for virtually every psychological, behavioral, and health problem across the lifespan [52]. Perhaps the most significant impact of poverty is its contribution to child abuse and neglect [53].

Stress associated with maltreatment compromises children’s immune systems and drives inflammatory processes that contribute to obesity, diabetes, cancer, and, ultimately, cardiovascular disease [54,55].

Further evidence that child poverty is a risk factor for ill health comes from policy research on the benefits of increased family income for example via the Earned Income Tax Credit and the 2021 Child Tax Credit. A working paper from the National Bureau of Economic Research concluded that permanent expansion of the Child Tax Credit “would cost $97 billion per year and generate social benefits with net present value of $982 billion per year.” Chapin Hall has amassed extensive evidence that economic and concrete supports for poor families significantly reduce the risk of child abuse and neglect [56].

In sum, allowing families to endure poverty directly contributes to health disparities, while policies that reduce child poverty engender beneficial effects on children’s health even into adulthood. Reduction in child poverty is a critically important and cost-effective prevention strategy that can produce a healthier population. Widespread poverty is largely due to laws and policies that were designed to increase corporate profits, as well as to advance a partisan agenda garnering political support from working class white people to the detriment of all in the working class [57].

## 6. Trump-Era Acceleration of Profit-First Governance

Recent federal policy provides a clear illustration of how a profit-maximization doctrine, when embedded in government decision-making, can directly undermine population health. The legislation popularly referred to as the *One Big Beautiful Bill* [58] is projected to reduce or eliminate health insurance coverage for approximately 17 million Americans over the coming decade—primarily through rollbacks of Medicaid eligibility, elimination of enhanced marketplace subsidies, and new administrative barriers to enrollment. Independent analyses estimate that these coverage losses would result in more than 50,000 additional premature deaths annually, consistent with prior evidence linking insurance loss to excess mortality.

These provisions were included to offset the cost of substantial tax reductions that overwhelmingly benefit high-income households. The Joint Committee on Taxation and multiple independent fiscal analyses estimate that roughly 80% of the bill’s tax benefits accrue to the top 1% of earners, including several hundred ultra-wealthy individuals who collectively receive tens of billions of dollars in reduced tax liabilities.

In effect, the federal government explicitly traded expanded access to essential health coverage—an intervention known to save lives—for fiscal gains concentrated among the highest-income Americans. This episode represents an unprecedented institutionalization of profit-first logic in federal health policymaking and underscores, in stark terms, how policies designed to maximize private financial benefit can generate predictable and preventable population-level harm.

## 7. Profit Maximization as a System of Selection by Consequences

Although prior empirical studies have shown that specific neoliberal policy instruments—such as privatization, deregulation, financialization, and reductions in social spending—are associated with adverse health outcomes, this research remains fragmented across sectors and rarely offers a unifying causal explanation. The present paper extends this literature by synthesizing evidence across health care, harmful-product industries, and social and economic policy to demonstrate that corporate profit maximization is the cross-cutting mechanism driving population-level harm. By documenting how profit-seeking behaviors are embedded in the regulatory, financial, and political arrangements that shape insurance markets, hospital ownership, pharmaceutical marketing, and structural inequalities, this paper provides an integrated account of the scale of U.S. health disparities and excess premature mortality.

It should be noted, however, that the relative influence of these three factors (i.e., profit-oriented clinical practices, deregulated marketing of harmful products, and corporate policy influence on mortality) on health are unknown. Such an analysis could guide the relative effort put into reforming policy in each of these areas.

To understand why harmful practices proliferate, we must recognize profit maximization as a system of selection by consequences. In behavioral science, selection by consequences refers to the process through which actions that produce reinforcement increase in frequency, while actions that do not are weakened or extinguished. This principle typically pertains to individuals but it readily applies as well to organizations and entire economic systems.

In the current U.S. political economy, profit is the dominant reinforcer. When a corporation engages in a practice—marketing addictive products to children, suppressing wages, denying needed care, manipulating pharmaceutical pricing, or designing digital platforms that amplify outrage—and that practice increases profit, the system reliably “selects” it for repetition, refinement, and expansion. This contingency is the engine driving many of the nation’s most serious health and social problems, including poverty, inequality, and preventable disease.

Profit maximization and selection by consequences are not limited to the U.S. Though beyond the scope of this article, it is likely that these processes are occurring in other countries which need to be studied with respect to global impact.

## 8. A Movement to Replace Profit-First Economics with Policies That Nurture Health and Wellbeing

The central problem we face is not corporate greed per se but the impunity with which corporations can engage in profitable, harmful practices. We show in this paper that such practices are endemic—not only in health care but in harmful-product industries and in the political strategies that allow wealthy individuals and corporations to shape laws to their advantage while harming millions of Americans.

To counter this, we need a broad social movement that establishes a new norm: business practices that harm people cannot be profitable. History provides precedents. The Progressive movement of the late 19th and early 20th centuries curbed extreme wealth concentration and reduced widespread corruption [59,60]. The New Deal further shifted power away from concentrated wealth by creating institutions—Social Security, Medicare, labor protections, anti-monopoly regulations—that formed the basis of the modern American middle class [61].

A second, opposing movement emerged in the mid-20th century. Propelled by the work of Milton and Rose Friedman [62] and funded by powerful interests [7], it worked over decades to normalize the idea that the unfettered pursuit of profit is the best path to prosperity and freedom. This movement shaped universities, media, think tanks, and political careers, gradually turning free-market ideology into the dominant framework for policymaking.

### 8.1. The Choice Before Us: Prosocial or Materialistic Values

At the same time that neoliberal beliefs and policies were being promoted, the behavioral and biobehavioral sciences converged on a very different conclusion: values and practices that promote the wellbeing of others are the foundation of thriving individuals and societies [57,63]. The pursuit of material wealth—beyond what is needed for basic security—fails to enhance wellbeing and often undermines it. By contrast, caring and cooperative environments reliably promote human flourishing.

This scientific consensus is the counterweight to free-market claims that prioritizing profit inevitably produces prosperity. Across all sectors, we must ask: To what extent is profit maximization contributing to this problem, and how would prioritizing human wellbeing change outcomes? Making this analysis explicit can unify groups working on health, education, environment, family policy, labor, and democracy.

### 8.2. A Single-Payer System

We believe that the movement to get a single-payer system, such as Medicare for All, is the best way to begin the reformation of the nation’s health care system. Our organization, *Values to Action*, is beginning an effort to expand this movement.

This may appear to be an insurmountable goal. Yet our review of the legislative landscape reveals that more than 100 Senators and Representatives have endorsed Medicare for All, and 27 national organizations have publicly supported this goal.

A social movement capable of achieving a single-payer system that guarantees Medicare for All would do far more than improve access to medical care. It would represent a fundamental shift in the societal contingencies that shape population health and wellbeing. By confronting one of the most entrenched expressions of profit-first economics, such a movement could catalyze a broader reorientation of public policy toward human flourishing, in multiple ways.

First, the establishment of a single-payer system would directly reduce morbidity and mortality by ensuring universal access to essential care, eliminating cost-related nonadherence, and reducing the administrative barriers that currently delay or prevent treatment. Evidence from other high-income nations demonstrates that when coverage is universal and health care financing is delinked from profit, preventable mortality falls, health disparities narrow, and life expectancy improves. In this sense, Medicare for All is not merely a health policy reform but a structural intervention that addresses one of the largest contributors to U.S. premature death.

Second, the movement required to secure such reform would build political capacities that extend far beyond health care. A successful campaign for Medicare for All demands cross-sector collaboration, widespread public engagement, and sustained pressure on elected officials—capacities that can subsequently be mobilized to address other systems in which profit maximization undermines wellbeing. By revealing the mechanisms through which financialized actors distort public institutions, the movement can help the public recognize similar patterns in housing, education, labor markets, environmental protection, and the marketing of harmful products.

Third, the adoption of a single-payer system would weaken the political influence of industries whose business models depend on extracting profit from human vulnerability. Reducing the power of private insurers, pharmaceutical monopolies, and hospital conglomerates would, in turn, challenge the broader architecture of corporate influence that shapes U.S. policy. This creates opportunities for reforms that address income inequality, reduce harmful-product marketing, strengthen social safety nets, and rebuild democratic institutions. In this way, health care reform becomes a gateway to a more comprehensive realignment of policy with the public interest.

Finally, a successful movement for Medicare for All would affirm a prosocial value framework that elevates care, equity, and collective responsibility over narrow materialism. Decades of research in behavioral and biobehavioral sciences show that societies grounded in these values exhibit higher levels of trust, cooperation, and psychological wellbeing. Thus, the movement would not only change policy but also help shift cultural norms toward prioritizing the wellbeing of all—a shift that is essential for tackling the multiple, interconnected crises that profit-driven systems have produced.

In sum, the pursuit of Medicare for All can be understood as both a health intervention and a democratic revitalization strategy. By challenging the dominance of profit maximization in one of the nation’s most consequential sectors, the movement has the potential to precipitate reforms across many domains, fostering a more nurturing society in which public policy consistently advances human wellbeing.

Clearly, the movement for Medicare for All exists, but its success hinges on a significant expansion of public support. Towards this goal and for the benefits articulated above, *Values to Action* has begun a project called “A Nurturing Nation”, which is initially focused on promoting Medicare for All, but with an eye toward addressing all the issues laid out in this article.

## Figures and Tables

**Table 1:** Policies Maintaining Poverty and Inequality.

Policy	Notes
Inadequate Minimum Wage	Increasing it would significantly reduce the number of people living in poverty [40]).
Restrictions on Union Organizing	The ability of workers to unionize has been constrained over the past forty years. When an industry unionizes, it increases not only pay of the unionized, but also the pay of those working in industries that must compete with unionized companies [41].
Monopolies increase the cost of cell phones, internet connections, numerous other commodities	Khan and Vaheesan [42] documented the degree to which free market theorists, jurists, and policy makers have eroded the regulation that was designed to prevent monopolies.
Incarceration of a large proportion of Black and Hispanic males	In 2018, the imprisonment of Black men was more than five and half times that of white men. This undermines family [43] income and stability and increases stress.
Tax system that minimizes taxes for the wealthy and corporations	The tax reform of 2025 adds $3.4 trillion to the federal deficit, while increasing the income of the wealthiest Americans and cutting social safety net programs like Medicaid and food assistance [44].
Forced mediation	The disputes with companies that stack the deck in favor of the companies [45].
Trade policies that have resulted in the loss of thousands of good paying jobs in the U.S.	700,000 U.S. jobs were lost due to the movement of production to other countries, where labor was cheaper [46].
Refusal by states to implement the ACA	Approximately 1.5 million Americans do not have health insurance [47]. It has been estimated that having health insurance reduces the risk of premature death by 0.71 to 0.97% [48].

## Data Availability

This is not applicable because the article is not based on a dataset.
